# How tree species identity and diversity affect light transmittance to the understory in mature temperate forests

**DOI:** 10.1002/ece3.3528

**Published:** 2017-11-11

**Authors:** Bram K. Sercu, Lander Baeten, Frieke van Coillie, An Martel, Luc Lens, Kris Verheyen, Dries Bonte

**Affiliations:** ^1^ Department of Biology Terrestrial Ecology Unit (TEREC) Ghent University Gent Belgium; ^2^ Department Forest and Water Management Forest & Nature Lab Ghent University Gontrode Belgium; ^3^ Lab Forest Management & Spatial Informat Tech Ghent University Gent Belgium; ^4^ Department Pathology, Bacteriology and Avian Diseases Ghent University Merelbeke Belgium

**Keywords:** canopy closure, resource heterogeneity, shrub and subcanopy layer, spatiotemporal variation, understory light

## Abstract

Light is a key resource for plant growth and is of particular importance in forest ecosystems, because of the strong vertical structure leading to successive light interception from canopy to forest floor. Tree species differ in the quantity and heterogeneity of light they transmit. We expect decreases in both the quantity and spatial heterogeneity of light transmittance in mixed stands relative to monocultures, due to complementarity effects and niche filling. We tested the degree to which tree species identity and diversity affected, via differences in tree and shrub cover, the spatiotemporal variation in light availability before, during, and after leaf expansion. Plots with different combinations of three tree species with contrasting light transmittance were selected to obtain a diversity gradient from monocultures to three species mixtures. Light transmittance to the forest floor was measured with hemispherical photography. Increased tree diversity led to increased canopy packing and decreased spatial light heterogeneity at the forest floor in all of the time periods. During leaf expansion, light transmittance did differ between the different tree species and timing of leaf expansion might thus be an important source of variation in light regimes for understory plant species. Although light transmittance at the canopy level after leaf expansion was not measured directly, it most likely differed between tree species and decreased in mixtures due to canopy packing. A complementary shrub layer led, however, to similar light levels at the forest floor in all species combinations in our plots. *Synthesis*. We find that a complementary shrub layer exploits the higher light availability in particular tree species combinations. Resources at the forest floor are thus ultimately determined by the combined effect of the tree and shrub layer. Mixing species led to less heterogeneity in the amount of light, reducing abiotic niche variability.

## 1 INTRODUCTION

Light availability at the forest floor is of central importance to many forest ecosystem processes. Light is an important resource affecting the performance and diversity of understory plants (Bartels & Chen, 2010; Bartemucci, Messier, & Canham, 2006; Jelaska, Antonić, Božić, Križan, & Kušan, 2006). It also has a large impact on the microclimatic conditions at the forest floor such as soil and air temperature and soil water content (Gray, Spies, & Easter, 2002; Ritter, Dalsgaard, & Einhorn, 2005). This microclimate shapes the diversity and composition of several organism groups, especially endothermic animals (Niemelä, Haila, & Punttila, 1996; Richards & Windsor, 2007), and influences ecosystem processes such as litter decomposition and both direct and indirect tree regeneration (Hobbie et al., 2006; Lin et al., 2014; Tingstad, Olsen, Klanderud, Vandvik, & Ohlson, 2015).

Light transmittance to the forest floor is spatially and temporally variable and is largely determined by tree species composition, stand density, stand structure, and canopy patterns, including the spatial arrangement of tree crowns and canopy gaps (Martens, Breshears, & Meyer, 2000; Tinya & Ódor, 2016). These aspects of forest structure change with developmental stage. Old‐growth forests with natural tree fall dynamics often show a high quantity and spatial heterogeneity of light transmittance (Canham, Finzi, Pacala, & Burbank, 1994; Tinya & Ódor, 2016), but only few of the temperate forests are actually in the old‐growth stage (Bengtsson, Nilsson, Franc, & Menozzi, 2000; Hannah, Carr, & Lankerani, 1995). The more prevalent mature forests in the understory re‐initiation stage generally have continuous closed canopies and light is expected to be primarily determined by species composition under similar stand basal area (Canham et al., 1994; Ligot, Ameztegui, Courbaud, Coll, & Kneeshaw, 2016). For these systems, it is therefore important to understand how the tree community composition (here identity and diversity) affects the light availability at the forest floor.

Several studies found that mixed stands had a denser canopy than monocultures because of complementary crown architecture and plasticity (Jucker, Bouriaud, & Coomes, 2015; Pretzsch, 2014; Sapijanskas, Paquette, Potvin, Kunert, & Loreau, 2014; Williams, Paquette, Cavender‐Bares, Messier, & Reich, 2017). Such increased canopy packing allows the trees to preempt the light resource more effectively and leads to lower light availability below the canopy (Forrester et al., 2017; Ligot et al., 2016). This is in accordance with the more general prediction of functional biodiversity research that more diverse systems use resources more efficiently due to complementarity between species (Forrester, 2014; Loreau & Hector, 2001; Tilman, 1999). Two experimental studies from tropic regions concluded that mixed stands of young trees had a higher light interception than any of the monocultures (Le Maire et al., 2013; Sapijanskas et al., 2014). Two different studies using a computer model of broadleaf and coniferous trees found a complementarity effect. Light interception in mixed stands was intermediate between the interception values in monocultures but higher than expected if the effects would be purely additive (Forrester et al., 2017; Ligot et al., 2016). The three mentioned studies focus on forests in the stem exclusion phase; however, evidence for higher light interception in more diverse forests from field studies in mature forests in the understory re‐initiation stage is lacking. In these systems, light interception might not occur solely by tree crowns but also on shrub level (Bartemucci et al., 2006; Messier, Parent, & Bergeron, 1998).

While mean light transmittance is predicted to decrease in mixed stands, it is unclear whether spatial heterogeneity of light transmittance will increase or decrease. More diverse forest stands are often assumed to create a more heterogeneous environment, because the trees create species‐specific conditions below their canopies (Ampoorter, Baeten, Koricheva, Vanhellemont, & Verheyen, 2014; Barbier, Gosselin, & Balandier, 2008; Vockenhuber et al., 2011). Reich, Frelich, Voldseth, Bakken, and Adair (2012) found that heterogeneity increased with decreasing light quantity on the stand level. Other studies found that variability in understory light peaked at 40% canopy cover (Martens et al., 2000) and decreased with increasing canopy cover (Dupré, Wessberg, Diekmann, & Lepš, 2002; Ligot et al., 2016). Variability of light transmittance would in this case decrease in mixed stands if canopy density is increased due to complementarity between species. Ligot et al. (2016) explicitly studied the effect of species mixtures on light heterogeneity and found mixed results depending on which species were used in the two models with highest stand basal area. Mixing pine and fir increased heterogeneity compared to pure stands, whereas other mixtures had intermediary levels of heterogeneity.

The light environment at the forest floor shows not only spatial but also temporal variability. Light conditions change dramatically throughout the season, especially in temperate deciduous forests. The total light transmittance throughout a year will be determined by the position of the sun, the amount and position of gaps before and after leaf expansion, and the timing of bud burst and leaf senescence. In temperate regions, the period in spring before leaf expansion can contribute disproportionately to the total biological relevant light reception at the forest floor. For example, tree saplings can receive more than 90% of the total annual irradiance before leaf expansion of adult trees (Augspurger, Cheeseman, & Salk, 2005). Substantial inter‐ and intraspecific variation in the timing of tree leaf expansion exists (Bobinac, Batos, Miljković, & Radulović, 2012; Lechowicz, 1984; Wesolowski & Rowinski, 2006), which could create large differences in the yearly biological relevant light transmitted to the forest floor between species compositions. This has never been studied in temperate forests as far as we know.

Here, we investigated how the quantity and spatiotemporal heterogeneity of light on the forest floor in mature temperate forests vary with tree identity and diversity. We used a tree diversity‐oriented research platform composed of deciduous tree species with different light transmittance characteristics. The tree species used in this study were (in order of increasing light transmittance) *Fagus sylvatica* L. (beech*), Quercus rubra* L. (red oak), and *Quercus robur* L.(pedunculate oak) (Canham et al., 1994; Ellenberg, 1988; Rebbeck, Gottschalk, & Scherzer, 2011). We quantified the shrub and canopy cover and measured light transmittance before, during, and after leaf expansion to answer the following questions: (1) How do tree identity and diversity determine tree canopy cover and shrub cover; (2) is the light quantity at the forest floor different between species and is it lower in mixtures than expected from monoculture values; (3) do monocultures differ in light heterogeneity and do mixtures decrease heterogeneity in light transmittance; (4) do the patterns in light quantity and heterogeneity differ between key phenological periods: before, during, and after leaf expansion?

## 2 MATERIALS AND METHODS

### 2.1 Site and experimental setup

The research was conducted across 53 plots (30 m × 30 m) located in mature forests in the region of Ghent, Belgium (the “TREEWEB” platform; Fig. S1; De Groote et al., in press). All forests have been historically continuously forested. The plots were selected to vary principally in tree species identity and diversity, while minimizing the variation in other environmental variables. The plots have similar soil texture and are mature, extensively managed forests that showed no signs of recent management. With a species pool of three regionally common tree species (*Quercus robur, Quercus rubra,* and *Fagus sylvatica),* a diversity gradient from monocultures to three species mixtures was created. Each of the seven possible species combinations was included in the design, with seven or eight realizations for each combination. During plot selection, the admixture of nontarget tree species was minimized (≤5% of the basal area) and the evenness of the target tree species in mixtures was maximized (>60% of maximum evenness based on basal area) (Baeten et al., 2013). The study plots had a mean stem number of 16 trees per plot (178 trees/ha; range: 100–333 trees/ha) and a mean basal area of 38.58 m²/ha (range: 25.09–52.48 m^2^/ha).

The three selected focal tree species represent different light strategies. They strongly differ in their light transmittance and shade tolerance, two tree characteristics that are generally inversely related (Canham et al., 1994). Based on studies about light transmittance and the shade tolerance ranks, we can assume that *F. sylvatica* has the lowest and *Q. robur* the highest light transmittance, while *Q. rubra* has an intermediate transmittance (Ellenberg, 1988; Niinemets & Valladares, 2006). *Q. rubra* is an exotic species but is abundant and economically important in the region, which makes it relevant to study. The status as exotic species is, however, expected to have no influence on light transmittance.

### 2.2 Tree and shrub cover

For each 900‐m² plot, we mapped the position of each tree with a diameter at breast height (DBH) larger than 15 cm using the Field‐Map system (www.field-map.com). For all the trees of which the crown covered part of the plot, we measured the dbh and crown projection to four directions. Each plot was subdivided into four 15 m × 15 m squares. Five subplots of 5 m × 5 m were established, one in the center of each square and one in the center of the plot. Shrub cover was visually estimated for each of the five subplots as the vertical projection of the shrub layer (Fig. S2). The shrub layer was defined as all shrub and subcanopy woody species between 1.5 and 7 m high. To analyze the data, the mean shrub cover per plot was calculated from the five subplot values. Total crown area per plot, the sum of all individual tree crown projections that fell within the plot area, was calculated based on crown projections in the Field‐Map system using QGIS (QGIS Development Team, 2009).

### 2.3 Quantifying light transmittance

**Figure 1:**
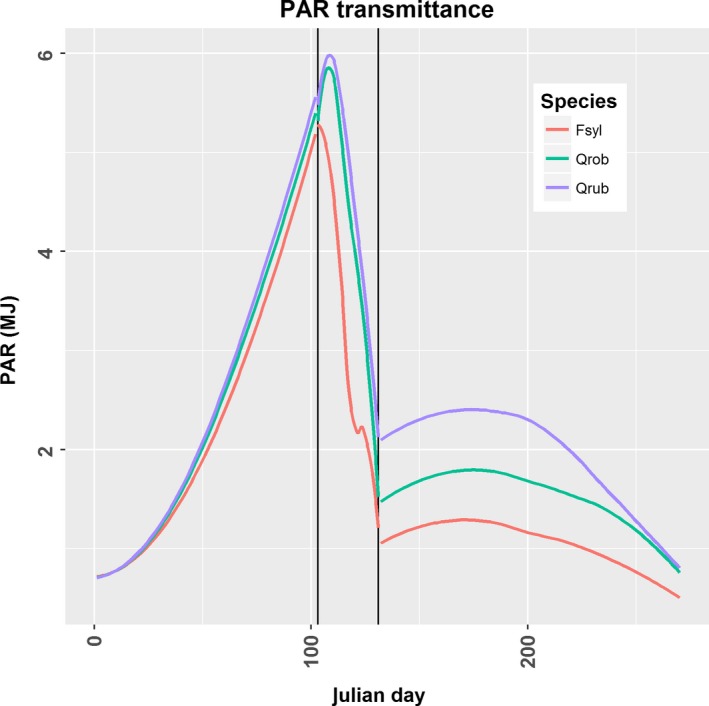
Loess smoother for the mean transmitted PAR for the three monocultures from 1 January until 12 October. The vertical lines at Julian days 103 and 131 enclose the period of leaf expansion

Light transmittance was measured with hemispherical images before and after leaf expansion at four locations in each of the 53 plots. The locations of the images were equally distributed over the plot, at least 5 m away from the edge of the plot and *ca*. 10 m of each other, avoiding spatial autocorrelation (Lin et al., 2003). The first series of images was captured between 18 and 20 March 2016, that is, well before budburst of any of the tree or shrub species in these communities. The second series of images was captured between 22 and 24 May 2016, that is, two weeks after full leaf expansion of all trees in the plots. For a subset of two randomly selected plots within each possible species combination along the diversity gradient (*N *=* *14), we additionally took images at the four locations per plot weekly, between the aforementioned dates. With this sampling, we obtained a time series of ten images per location covering the entire leafing‐out period. Hemispherical images were taken at 1.5 m height, with the top of the camera orientated north. We used a Nikon D5200 (24.1 megapixels, dynamic range of 12.5 Ev at ISO 200) with a circular fisheye lens (Sigma EX 4.5 mm) fixed in a self‐leveling mount on a tripod to obtain a horizontal position of the lens. Images (6,000 × 4,000 pixels, 14 bit/color, ISO 200) were taken when sky illumination was homogeneous, that is, during overcast days or during an interval of 90 minutes centered around sunrise or sunset. Histogram selection based on Beckschäfer, Seidel, Kleinn, and Xu (2013) was too time‐consuming to perform in the field. Instead, three images with different underexposure (−3 ± 1.3 stops) using matrix light measurement were taken at each location using the bracketing function (Beckschäfer et al., 2013; Brusa & Bunker, 2014). We automatically selected the image with the highest exposure value but with fewest overexposed pixels for further analysis. Binarization of the images was performed with the K‐means clustering algorithm (Lloyd's algorithm) from the “scikit‐learn.cluster” package in python (Pedregosa et al., 2011) as this was one of the best‐performing algorithms according to the review by Jonckheere, Nackaerts, Muys, and Coppin (2005). We used two clusters, other parameters where kept at default values. We used the free software CIMES (Gonsamo, Walter, & Pellikka, 2011) to calculate total transmitted PAR to the forest floor, both direct and diffuse radiation, based on the binarized images. We calculated total transmitted PAR for standard overcast conditions (SOC) and for clear sky condition (CLEAR) (Figure 1). Based on these estimates, we calculated the gap light index (GLI), which is the total transmitted PAR to the forest floor as a percentage of total incident PAR above the canopy (Canham, 1988)

(1) GLI=[(Tsoc∗Psoc)+(Tclear∗Pclear)]∗100

Psoc and Pclear are the proportions of days with overcast sky and clear sky conditions, respectively. Tsoc and Tclear are the proportions of diffuse and direct‐beam radiation that are transmitted through the canopy to the understory under overcast sky and clear sky conditions, respectively. A GLI of 0 indicates that there is no light in the understory, while a GLI of 100 indicates a totally open site.

For the calculation of GLI, the parameters Psoc and Pclear were estimated as 0.5 which is an average value for Western Europe for the entire growing season that approximates our local conditions (Gendron, Messier, & Comeau, 1998). Tsoc and Tclear are calculated for each day using CIMES based on the appropriate hemispherical image. We calculated one GLI for each of the three periods: before (01 January–12 April), during (13 April–9 May), and after (10 May–12 October) leaf expansion based on the images captured in the respective time periods. The GLI during leaf expansion was only calculated for the fourteen plots that were measured weekly. Julian day 286, 12 October, was considered as the end of the growing season and this date coincides with the onset of leaf coloration in trees (Gressler, Jochner, Capdevielle‐Vargas, Morellato, & Menzel, 2015).

### 2.4 Statistical analyses

To investigate how the identity and diversity of the tree species affect the tree and shrub cover, we calculated a set of diversity–interaction models with either shrub cover per plot or total crown area per plot as dependent variable (Kirwan et al., 2009). A first null model (M0) assumes that all tree species have a similar, noninteractive effect on the dependent variable. The dependent variable is modeled in function of total basal area of all trees in the plot and one intercept is estimated. The species identity model (M1) models the dependent variable as a function of total basal area and the relative abundance (based on basal area) of each focal tree species. This model estimates a species‐specific intercept, but does not allow for species interaction effects in mixtures, assuming purely additive effects. The species interaction model (M2) extends M1 by adding the two‐way and three‐way species interactions between the species’ relative abundances. The model thus estimates a species‐specific intercept (identity effect) and the interactions between species’ relative abundances (diversity effect), while accounting for total basal area. The diversity effect (interaction) is then the difference between the actual performance of a mixture and the performance expected from the monoculture performances. The total crown area was modeled with a Gaussian distribution and shrub cover, which was bound between 0 and 1, was modeled with a beta distribution. All analyses were performed in the probabilistic programming language Stan, called from R using the package brms in R 3.3.0 (Buerkner, 2016; R Core Team, 2016). We used the widely applicable information criterion, WAIC (Vehtari, Gelman, & Gabry, 2017), to compare the models and identify the most parsimonious model that best explains the data.

To study the intertwined effect of species identity and species diversity on the spatial and temporal variation in light availability, we modeled the GLI with a species interaction model similar to M2 specified above. We extended the model proposed by Kirwan et al. (2009) by including “plot” as a random effect to account for the spatial dependence of the variables measured at the four locations within plots. Furthermore, rather than having a single residual variance term to quantify the within‐plot variation in GLI, this variance was allowed to be different for each species composition level. This implies that the within‐plot variance is a relative measure for heterogeneity for each composition, an important variable in addition to the mean light quantity to understand the light environment in forests. Such model thus allows quantifying differences in within‐plot light variation between the different species combinations and diversity levels. Models were again fitted using brms. We ran the model for the three light variables: GLI before, during, and after leaf expansion. All three dependent variables are bound between 0 and 1; therefore, a beta distribution was used.

## 3 RESULTS

### 3.1 Effect of tree identity and diversity on tree crown and shrub cover

**Figure 2:**
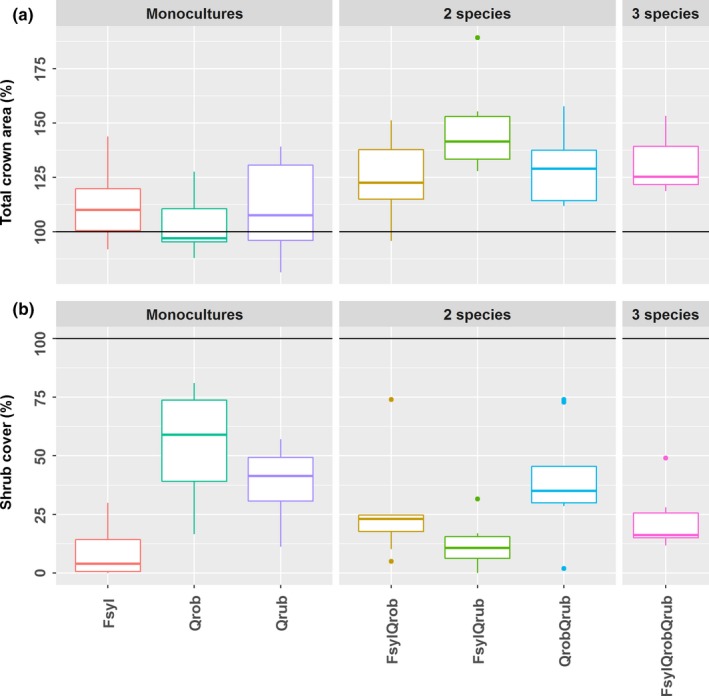
Boxplots of (a) total crown area and (b) shrub cover as a percentage of the plot area for each species combination. The horizontal black line indicates 100%

The model that best explained the total crown area was the species interaction model (M2) which means that there were significant interaction effects when species grow in mixtures (Table 1). Total tree crown area was significantly higher in all mixtures than expected based on monoculture values. The null model had a slightly lower WAIC value than the identity model which means that the monoculture values for total crown area were similar. Although the *Q. robur* monoculture had the lowest and *Q. rubra* the highest total crown area, these differences were not significant as the parameter estimates for the monoculture values have a large overlap (Figure 2; Table S1).

**Table 1:** WAIC (widely applicable information criterion) values for the three nested models for the two dependent variables. Total crown area is the sum of all tree crown areas, and shrub cover is the mean of the estimated shrub cover for the five subplots. The lowest WAIC, thus the best model, is indicated in bold

	Total crown area	Shrub cover
M0: null model	477.09	−27.47
M1: species identity model	478.74	**−56.15**
M2: species interaction model	**463.67**	−52.55

Shrub cover was best explained by the more parsimonious species identity model (M1) (Table 1.) because it differed between tree species. The shrub cover under *F. sylvatica* monocultures was lower than under monocultures of *Q. rubra* and *Q. robur*. The shrub cover in mixtures did not differ from the expected cover based on monocultures and interactions were not significant, which means that shrub cover was explained only by additive effects (Figure 2; Table S1). Overall, we observed a large variation in shrub cover, and plot values ranged from 0% to 82%.

### 3.2 Identity effect on light transmittance before, during, and after leaf expansion

**Figure 3:**
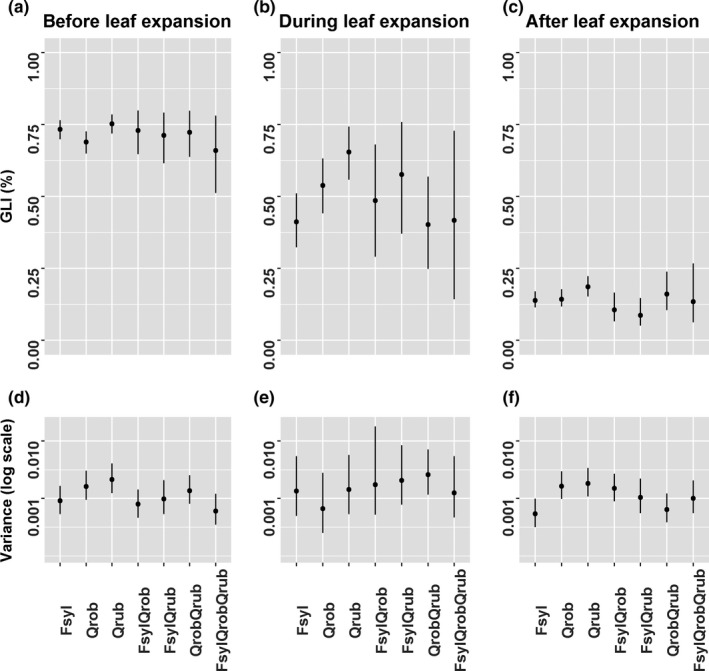
Estimates of the light transmittance (GLI) with 95% credible intervals for monocultures and the different mixtures (based on posterior parameter estimates of the species interaction model). Equal basal area of the species is assumed. Variances are calculated using the estimate for the GLI for each species combination. (a) Mean GLI before, (b) mean GLI during, (c) mean GLI after leaf expansion and (d) variance of GLI before, (e) variance of GLI during, (f) variance of GLI after leaf expansion

Mean light transmittance in monocultures did not significantly differ between any of the species neither before nor after leaf expansion. Mean GLI in the monocultures ranged from 68.90% (*Q. robur*) to 75.29% (*Q. rubra*) before leaf expansion and decreased after leaf expansion to a range from 14.57% (*F. sylvatica*) to 18.91% (*Q. rubra*). The GLI during leaf expansion did differ significantly between all monocultures (Figure 3; Table S2).

Before leaf expansion, the three monocultures had a similar within‐plot variance of GLI although variance of *F. sylvatica* tends to be somewhat lower. During leaf expansion, within‐plot variance is similar for all species combinations. The posterior values have large credibility intervals due to the low sample size during leaf expansion. After leaf expansion, the within‐plot variance in *F. sylvatica* monocultures was significantly lower compared to *Q. robur* and *Q. rubra* monocultures (Figure 3; Table S3).

### 3.3 Diversity effect on light transmittance before, during, and after leaf expansion

Most mixtures showed no diversity effects in any of the time periods and had a GLI which was purely determined by additive effects, that is, intermediate between the monoculture values. Diversity effects on mean GLI were only observed in mixtures of *Q. robur*–*Q. rubra* during leaf expansion and *F. sylvatica*–*Q. rubra* after leaf expansion. These mixtures had a significantly lower light transmittance than expected based on monocultures (Figure 3; Table S2).

The within‐plot variance of GLI before, during, and after leaf expansion was always intermediate between or lower than the constituent monocultures. The three species mixtures before leaf expansion showed lower within‐plot variance than we would estimate from the monocultures. Within‐plot variance during leaf expansion is similar for all species combinations. The *Q. robur*–*Q. rubra* mixture after leaf expansion had a lower within‐plot variance than we would estimate from the monocultures. Within‐plot variance in mixtures before and after leaf expansion was never higher than highest within‐plot variance in monocultures (Figure 3; Table S3).

## 4 DISCUSSION

We measured light transmittance across a tree diversity gradient of mature forest plots to study how light quantity and heterogeneity at the forest floor are influenced by the identity and diversity of the trees throughout the year. Light conditions will ultimately be determined by the combined interception of tree and shrub cover. Therefore, we additionally measured tree identity and diversity effects on the tree and shrub cover. While the three different tree species showed similar total crown area, this crown area increased in mixtures. This positive diversity effect is in agreement with earlier studies (Jucker et al., 2015; Pretzsch, 2014; Williams et al., 2017). Contrary to the total crown area, however, we observed a clear identity effect but no diversity effects on shrub cover. *F. sylvatica* had a lower shrub cover compared with both *Q. robur* and *Q. rubra*. This is in line with the lower cover of the herb layer community below beech found in other studies (Mӧlder, Bernhardt‐Rӧmermann, & Schmidt, 2008; Wulf & Naaf, 2009).

We found that the quantity of light transmitted before and after leaf expansion is similar in all species compositions and thus little impacted by identity or diversity effects. During the period of leaf expansion we found, however, a clear identity effect and all three monocultures differed significantly in the quantity of transmitted light. These differences in spring light transmittance are anticipated to have a great impact on yearly biological relevant light availability for understory plants.

In terms of light heterogeneity after leaf expansion, we found that the level of variation is species‐dependent: In *F. sylvatica* monocultures, light is much more homogeneously distributed compared with the two *Quercus* species. Increased tree diversity does not lead to an increased heterogeneity and heterogeneity is actually intermediate or lower than that of the constituent species. While mixtures have an increased tree crown cover compared to monocultures, this did not lead to a lower light transmittance to the forest floor in mixtures, most likely due to complementary light interception by the shrub layer and a high variability in GLI within plots.

### 4.1 Identity effect on light quantity and heterogeneity

The light transmission before leaf expansion was similar across species combinations which is not surprising as light interception of stems and branches is expected to be similar between tree species. We did find that monocultures significantly differ from each other in light transmittance during the 3 weeks of leaf expansion. Light transmittance was lowest in *F. sylvatica* monocultures, highest in *Q. rubra* monocultures, and intermediate in *Q. robur*. These differences might be due to differences in timing of leaf expansion between the species. The dense shrub layer of the early leafing‐out *Coryllus avellana* under *Q. robur* caused lower light transmittance than would be expected based solely on the tree layer.

Contrary to our expectations and to other studies (Canham et al., 1994; Härdtle, von Oheimb, & Westphal, 2003; Vockenhuber et al., 2011), we did not find differences in light transmittance to the forest floor after leaf expansion between *Q. robur*,* F. sylvatica,* and *Q. rubra* monocultures. We attribute this lack of tree identity signal on light transmittance to the varying contribution of the shrub layer. In monocultures of *F. sylvatica*, there was almost no shrub cover while monocultures of *Q. robur* had a very high shrub cover and monocultures of *Q. rubra* had an intermediate shrub cover. In the virtual absence of a shrub layer, the low light transmittance in *F. sylvatica* was purely determined by the dense tree canopy. In *Q. robur* stands, on the other hand, the combined light interception by the tree and the abundant shrub layer was similar to *F. sylvatica*, which implies that interception by the *Q. robur* canopy was lower than that of *F. sylvatica*. These results are similar to the results of Bartemucci et al. (2006) who found uniformly low light levels at the lower understory and forest floor level despite clear differences in light transmittance above the shrub layer at a height of 4 m.

Almost all studies looking at tree species effect on understory processes solely measure light transmittance after leaf expansion. Light transmittance in early spring, before and during leaf expansion, is, however, as important or even more important for understory plant growth (Augspurger & Salk, 2017; Augspurger et al., 2005; Baeten, Sercu, Bonte, Vanhellemont, & Verheyen, 2015). Augspurger et al. (2005) found that seedlings of different tree species received between 33 and 97.6% of their total irradiance before 100% canopy closure. Small differences in timing of leaf expansion between trees could therefore lead to large differences in total irradiance. Although it is generally acknowledged that canopy avoidance in forest herbs and seedlings is ubiquitous, differences in light transmittance during this period are almost never accounted for when studying the effect of tree species on understory cover, diversity, and other light‐dependent processes.

Despite the similar mean light quantity after leaf expansion in the different species compositions, the light heterogeneity in *F. sylvatica* is significantly lower than in the two *Quercus* monocultures. Our observation that *F. sylvatica* has a homogeneous low light transmittance and an early leaf expansion, while the shrub layer is almost absent, confirms other observations that find low light transmittance in *F. sylvatica* stands (Härdtle et al., 2003; Mӧlder et al., 2008; Vockenhuber et al., 2011).

### 4.2 Diversity effect on light quantity and heterogeneity

Our expectation that mixtures would show an increased canopy packing due to complementarity effects was confirmed as total tree crown area was higher than expected based on the monoculture values for all mixtures. The mixture of *F. sylvatica*–*Q. rubra* showed the highest increase in crown area.

An increased tree diversity has no effect on light transmittance before canopy closure and leads to intermediate light transmittance during leaf expansion for *F. sylvatica*–*Q. rubra* and *F. sylvatica*–*Q. robur* mixtures. The mixture *Q. robur*–*Q. rubra* had a lower light transmittance during the period of leaf expansion than would be expected based on monoculture values. The lower light transmittance in the mixture of *Q. robur*–*Q. rubra* is probably an artifact due to a relative low light transmittance before leaf expansion and an early leaf expansion in this particular subset of two plots that was used to determine light transmittance during leaf expansion.

Based on the increased canopy packing, we expected that light transmittance through the canopy will decrease in all mixtures after leafing out. At the forest floor, however, there was only a significant diversity effect in the *F. sylvatica*–*Q. rubra* mixture. This mixture had a high increase in crown area. Moreover, differences in light transmittance between this mixture and the monocultures are purely the effect of tree canopy density as shrub cover is very low in the mixture and monocultures of *F. sylvatica* and *Q. rubra*. The other two mixtures had a similar light transmittance after leaf expansion at the forest floor compared to the monocultures. This is most likely due to the complementary shrub layer that was high in plots with a rather open canopy and lower under closed canopies. In conclusion, we do find indication for a higher light interception at the tree canopy level if tree diversity increases (Jucker et al., 2015; Ligot et al., 2016), but the complementary shrub layer effectively homogenizes light transmittance to the forest floor across species combinations.

Higher tree diversity decreased within‐plot heterogeneity to levels intermediate or lower than expected based on monoculture values. This contradicts with the assumption that tree diversity will create more heterogeneous conditions on the forest floor which is implicitly or explicitly made in many studies (Ampoorter et al., 2016; Dupré et al., 2002; Reich et al., 2012). From the perspective of *F. sylvatica* monocultures, however, adding other species breaks the homogeneous light transmittance and increases heterogeneity (Härdtle et al., 2003; Mӧlder et al., 2008; Vockenhuber et al., 2011).

### 4.3 Impacts for understory plants

The importance of light quantity and heterogeneity for processes at the forest floor is well studied with regard to plant understory diversity and cover (Mӧlder et al., 2008; Thomsen, Svenning, & Balslev, 2005; Tinya, Márialigeti, Király, Németh, & Odor, 2009; Tinya & Ódor, 2016). Increased tree diversity in stands is generally assumed to create a higher heterogeneity of abiotic conditions at the forest floor (Ampoorter et al., 2016; Canham et al., 1994; Dupré et al., 2002; Thomsen et al., 2005) and the resulting higher number of niches in the mixed stand is expected to promote the coexistence of more understory species (Barbier et al., 2008; Levine & HilleRisLambers, 2009; Reich et al., 2012). In young and mature stands that do not exhibit strong canopy dynamics, average light quantity also is an important factor in governing understory diversity and species composition (Bartels & Chen, 2010; Reich et al., 2012; Tinya et al., 2009).

Our results indicate that increasing tree diversity by intermixing species leads to lower heterogeneity within in forests in the understory re‐initiation stage. This might partially explain why several studies find no effect of overstory diversity on herbaceous diversity (Ampoorter et al., 2014, 2016; Both et al., 2011; Ewald, 2002; Gazol & Ibáñez, 2009; Houle, 2007; Thomsen et al., 2005). The study of Thomsen et al. (2005) suggests that the fine‐grained mixture of tree species attenuates the differential impact of the tree species on any given area on the forest floor by causing a mixing of their light and litter effects. Additionally, our results show that the shrub layer is able to attenuate the differential impact of the tree species on the forest floor leading to similar light quantities across different species combinations. Studies focusing on the effect of tree species identity and diversity on plant understory dynamics should thus include those components of the forest ecosystem that respond to tree species composition and determine the ultimate availability of resources.

## 5 CONCLUSION

Our results show that light transmittance to the forest floor after leaf expansion is similar across species combinations. Although light transmittance is expected to differ between tree species and to decreases in mixtures, a complementary shrub layer exploits the higher light availability in particular tree species combinations so that the ultimate light levels at the forest floor are similar across all species combinations in our plots. We found evidence that light transmittance during the 3 weeks of leaf expansion does, however, differ significantly between species. This could be a major source of variation in light transmittance between tree species with a large impact on the performance of understory plants. Finally, we show that in the case of light, higher tree diversity does not lead to higher heterogeneity of resources at the forest floor.

## AUTHOR'S CONTRIBUTIONS

Luc Lens, Kris Verheyen, Dries Bonte, An Martel, and Lander Baeten designed the TREEWEB platform and the methodology for the platform and this paper. Frieke Van Coillie gave critical input for designing the methodology for the hemispherical image acquisition and processing. Bram Sercu collected the data. Bram Sercu and Lander Baeten analyzed the data. Bram Sercu led the writing of the manuscript. All authors contributed critically to the drafts and gave final approval for publication.

## CONFLICT OF INTEREST

None declared.

## DATA ACCESSIBILITY

Tree and shrub cover data and light availability data: DRYAD entry doi:10.5061/dryad.8pv03.
