# Supplementary material for: How tree species identity and diversity affect light transmittance to the understory in mature temperate forests
Source: Ecol Evol. 2017 Nov 11;7(24):10861–70. doi: 10.1002/ece3.3528 (PMC5743659; doi:10.1002/ece3.3528)
Supplement: Supplementary file 1 [file ECE3-7-10861-s001.docx]

SUPPORTING INFORMATION


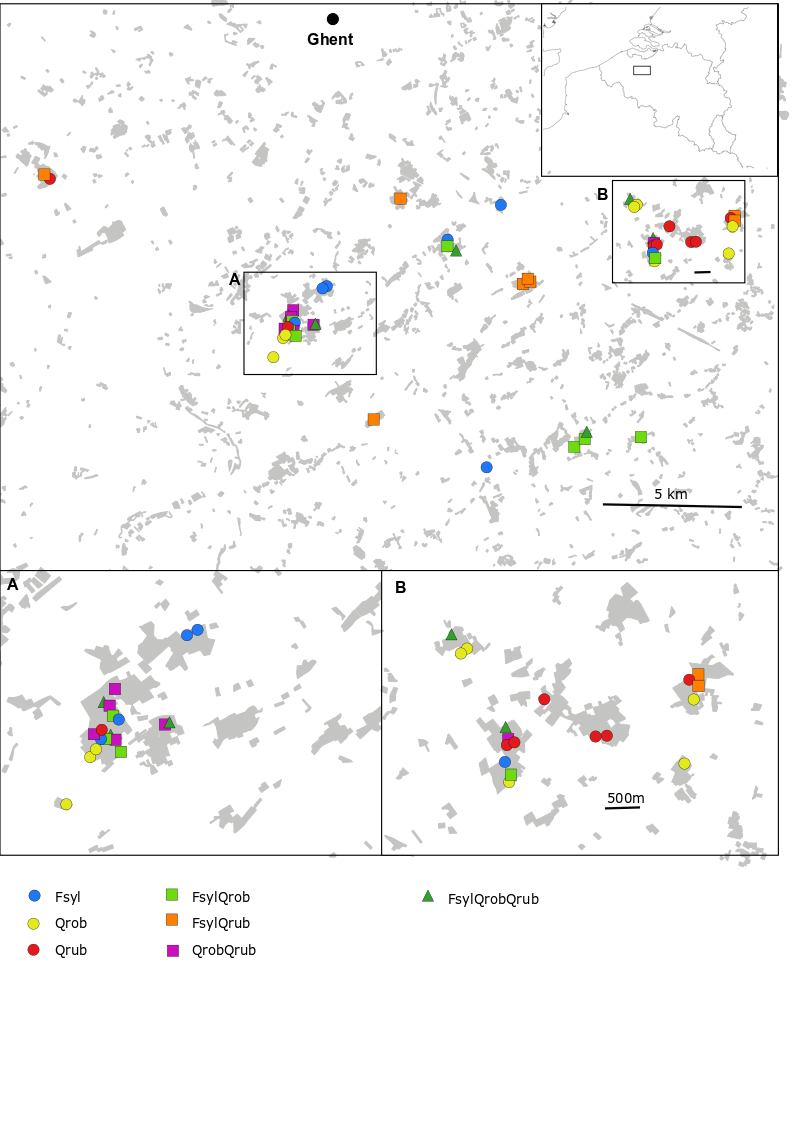


**Figure S1 --** Map of the study area in northern Belgium. Grey patches represent forest fragments, the 53 TREEWEB plots are indicated by coloured symbols, showing plot-level tree species diversity and composition (*Quercus robur* = Qrob, *Fagus sylvatica* = Fsyl and *Quercus rubra* = Qrub).

**
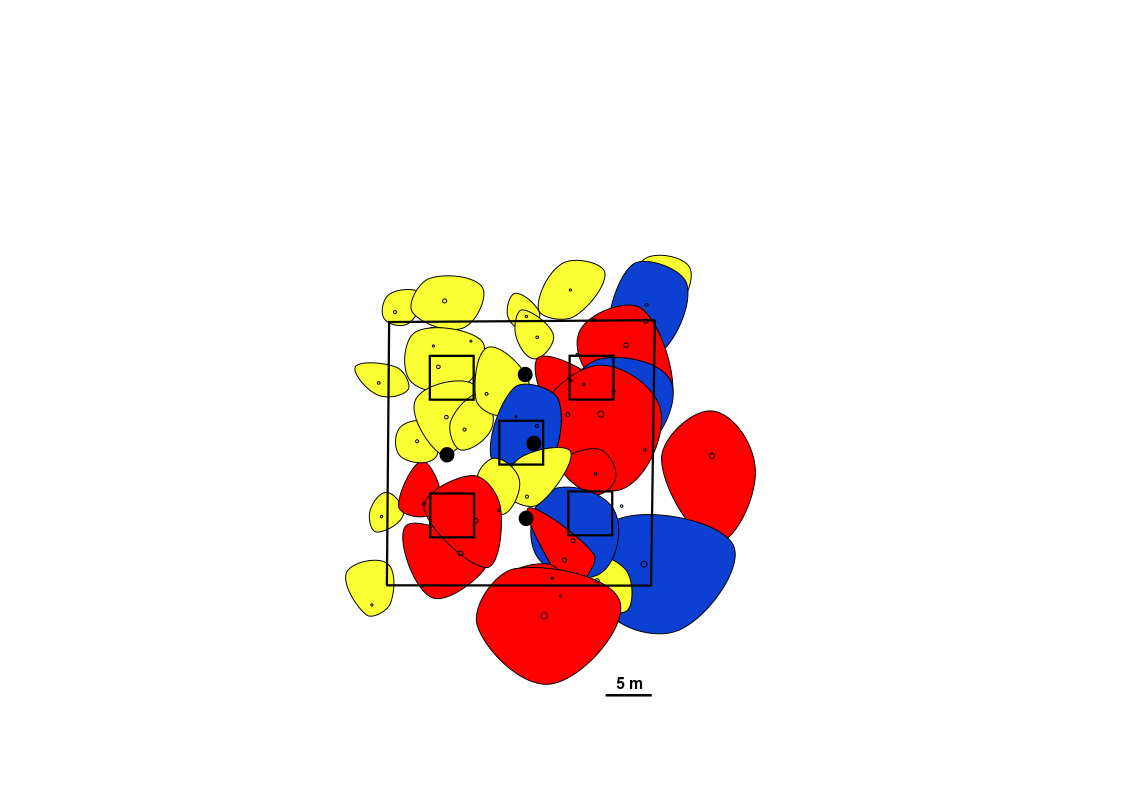
**

**Figure S2 --** A detailed plot map. The big square is the 30 m x 30 m plot, open circles represent tree stems, polygons are tree crown projections (different colours for different tree species). Small squares indicate the 5 m x 5 m subplots in which shrub cover was estimated, full circles are the locations where hemispherical images were taken.

**Table S1**: Posterior distribution of the parameter estimates (mean and 95% credible intervals between brackets) for the best model for total crown area and shrub cover. The best model for total crown area was the species interaction model (M2) and the identity model (M1) for shrub cover (see table 1 in M.S.). The colon between the species names indicates the interaction effect between relative basal area of Fsyl, Qrob and Qrub. Note that the parameter values for shrub cover are based on the logit transformed shrub cover values.

|  |  |  |  |
| --- | --- | --- | --- |
|  | | | |
|  | explanatory variable | total crown area | shrub cover |
|  | | | |
|  | Fsyl | 79.38 ( 43.07 - 115.90 ) | -2.38 ( -4.27 - -0.43 ) |
|  | Qrob | 75.02 ( 42.06 - 108.36 ) | 0.28 ( -1.52 - 2.02 ) |
|  | Qrub | 85.58 ( 51.87 - 120.67 ) | -0.77 ( -2.65 - 1.10 ) |
|  | Total basal area | 8.40 ( -1.16 - 17.82 ) | -0.01 ( -0.52 - 0.50 ) |
|  | Fsyl:Qrob | 76.55 ( 11.28 - 140.70 ) |  |
|  | Fsyl:Qrub | 142.71 ( 73.24 - 211.76 ) |  |
|  | Qrob:Qrub | 79.15 ( 16.05 - 144.24 ) |  |
|  | Fsyl:Qrob:Qrub | -319.09 ( -823.41 - 190.65 ) |  |
|  | | | |

**Table S2**: Posterior distribution of the parameter estimate (mean and 95% credible interval between brackets) for all explanatory variables in the species interaction model (M2) for GLI before, after and during leaf expansion. The colon between the species names indicates the interaction effect between relative basal area of Fsyl, Qrob and Qrub. Note that the parameter values for GLI are based on the logit transformed values of the GLI values.

|  | | | | | |
| --- | --- | --- | --- | --- | --- |
|  | explanatory variable | before leaf expansion | during leaf expansion | after leaf expansion |  |
|  | | | | | |
|  | Fsyl | 1.01 ( 0.84 - 1.18 ) | -0.35 ( -0.74 - 0.04 ) | -1.82 ( -2.05 - -1.59 ) |  |
|  | Qrob | 0.79 ( 0.61 - 0.97 ) | 0.16 ( -0.24 - 0.54 ) | -1.79 ( -2.02 - -1.53 ) |  |
|  | Qrub | 1.12 ( 0.94 - 1.29 ) | 0.64 ( 0.23 - 1.06 ) | -1.48 ( -1.71 - -1.25 ) |  |
|  | Total basal area | 0.02 ( -0.05 - 0.09 ) | -0.14 ( -0.32 - 0.04 ) | -0.03 ( -0.13 - 0.06 ) |  |
|  | Fsyl:Qrob | 0.17 ( -0.72 - 1.08 ) | 0.07 ( -1.85 - 1.93 ) | -0.66 ( -1.85 - 0.55 ) |  |
|  | Fsyl:Qrub | -0.33 ( -1.29 - 0.65 ) | 0.32 ( -1.65 - 2.23 ) | -1.41 ( -2.72 - -0.06 ) |  |
|  | Qrob:Qrub | 0.02 ( -0.9 - 0.96 ) | -1.59 ( -3.25 - -0.01 ) | -0.04 ( -1.2 - 1.12 ) |  |
|  | Fsyl:Qrob:Qrub | -2.55 ( -9.52 - 4.25 ) | -2 ( -16.81 - 12.59 ) | 2.71 ( -6.21 - 12.24 ) |  |
|  | | | | | |

| **Table S3**: Estimates of the within-plot variance of light transmittance (GLI), before, during and after leaf expansion, with 95% credible intervals for all species combinations. Variances are calculated using the estimate of the GLI for each species combination based on the posterior parameter estimates from the species interaction model (M2). Equal basal area of the species is assumed. | | | | | |
| --- | --- | --- | --- | --- | --- |
|  | species combination | before leaf expansion | during leaf expansion | after leaf expansion |  |
|  | | | | | |
|  | Fsyl | 5.36 ( 4.77 - 5.90 ) | 5.13 ( 3.79 - 6.19 ) | 5.43 ( 4.84 - 5.97 ) |  |
|  | Qrob | 4.88 ( 4.26 - 5.42 ) | 5.87 ( 4.49 - 6.90 ) | 4.33 ( 3.73 - 4.84 ) |  |
|  | Qrub | 4.46 ( 3.83 - 5.02 ) | 5.01 ( 3.68 - 6.04 ) | 4.21 ( 3.60 - 4.74 ) |  |
|  | FsylQrob | 5.51 ( 4.92 - 6.06 ) | 4.77 ( 2.64 - 6.18 ) | 4.41 ( 3.83 - 4.94 ) |  |
|  | FsylQrub | 5.32 ( 4.59 - 5.95 ) | 4.74 ( 3.37 - 5.75 ) | 4.77 ( 4.02 - 5.41 ) |  |
|  | QrobQrub | 4.98 ( 4.37 - 5.52 ) | 4.50 ( 3.53 - 5.33 ) | 5.25 ( 4.62 - 5.78 ) |  |
|  | FsylQrobQrub | 5.90 ( 5.22 - 6.46 ) | 5.21 ( 3.80 - 6.25 ) | 4.80 ( 4.10 - 5.42 ) |  |
|  | | | | | |
